# Effect of Antibiotics against *Mycoplasma sp.* on Human Embryonic Stem Cells Undifferentiated Status, Pluripotency, Cell Viability and Growth

**DOI:** 10.1371/journal.pone.0070267

**Published:** 2013-07-30

**Authors:** Leonardo Romorini, Diego Ariel Riva, Carolina Blüguermann, Guillermo Agustin Videla Richardson, Maria Elida Scassa, Gustavo Emilio Sevlever, Santiago Gabriel Miriuka

**Affiliations:** Laboratorio de Biología del Desarrollo Celular, Laboratorios de Investigación Aplicada en Nuerociencias - Fundación para la Lucha contra las Enfermedades Neurológicas de la Infancia, Escobar, Buenos Aires, Argentina.; Universidade de São Paulo, Brazil

## Abstract

Human embryonic stem cells (hESCs) are self-renewing pluripotent cells that can differentiate into specialized cells and hold great promise as models for human development and disease studies, cell-replacement therapies, drug discovery and *in vitro* cytotoxicity tests. The culture and differentiation of these cells are both complex and expensive, so it is essential to extreme aseptic conditions. hESCs are susceptible to *Mycoplasma sp.* infection, which is hard to detect and alters stem cell-associated properties. The purpose of this work was to evaluate the efficacy and cytotoxic effect of Plasmocin^TM^ and ciprofloxacin (specific antibiotics used for *Mycoplasma sp.* eradication) on hESCs. *Mycoplasma sp.* infected HUES-5 884 (H5 884, stable hESCs H5-brachyury promoter-GFP line) cells were effectively cured with a 14 days Plasmocin^TM^ 25 µg/ml treatment (curative treatment) while maintaining stemness characteristic features. Furthermore, cured H5 884 cells exhibit the same karyotype as the parental H5 line and expressed GFP, through up-regulation of brachyury promoter, at day 4 of differentiation onset. Moreover, H5 cells treated with ciprofloxacin 10 µg/ml for 14 days (mimic of curative treatment) and H5 and WA09 (H9) hESCs treated with Plasmocin^TM^ 5 µg/ml (prophylactic treatment) for 5 passages retained hESCs features, as judged by the expression of stemness-related genes (TRA1-60, TRA1-81, SSEA-4, Oct-4, Nanog) at mRNA and protein levels. In addition, the presence of specific *markers* of the *three germ layers* (brachyury, Nkx2.5 and cTnT: mesoderm; AFP: endoderm; nestin and Pax-6: ectoderm) was verified in *in vitro* differentiated antibiotic-treated hESCs. In conclusion, we found that Plasmocin^TM^ and ciprofloxacin do not affect hESCs stemness and pluripotency nor cell viability. However, curative treatments slightly diminished cell growth rate. This cytotoxic effect was reversible as cells regained normal growth rate upon antibiotic withdrawal.

## Introduction

Human embryonic stem cells (hESCs) are pluripotent cells derived from the inner cell mass of early human embryos. Under optimal *in vitro* culture conditions, these cells can self-renew and be cultured indefinitely in an undifferentiated state while maintaining stemness features. Moreover, they can differentiate into virtually all adult cell type derived from the three germ layers: ectoderm, mesoderm and endoderm (pluripotency). Therefore, hESCs hold great promise as models for human development and disease, as well as for drug discovery and cell-replacement therapies. Particularly, due to their reliance on many key pathways in morphogenesis and differentiation, hESCs may find an immediate pharmacological application for *in vitro* drug toxicity testing models [1–3].

hESCs *in vitro* culture maintenance and differentiation protocols are very expensive and time consuming processes. It is extremely important then, for both basic research and biotechnological manufacture, to avoid or eventually eradicate any type of microorganism contamination, like fungal or bacterial infections, from hESCs cultures. In particular, mycoplasmas are small microorganisms (0.3-0.8 µm) which lack a rigid cell wall and belong to the class Mollicutes (*Mycoplasma sp.* and *Acholeplasma* *sp.*) [4]. They are part of the physiological human flora as well as opportunistic pathogens. Moreover, *Mycoplasma sp.* is one of the most frequent contaminants found in eukaryotic cell cultures. In fact, their infection frequency ranges from 5 to 35% of cell cultures, depending upon the country and laboratory of origin [5,6], and it may be as high as 65-80% in some cell culture facilities [7]. *Mycoplasmas* within individual cell cultures could reach titers of 10^8^ colony forming units per milliliter [5]. Recent studies found that *Mycoplasma sp.* is one of the most common microbiological contaminants of stem cell cultures, as 4% (n=7) out of 158 cell passages from 32 stem cell and feeder cell lines were infected [8].

*Mycoplasmas* cannot be visualized under inverted microscope and neither show turbidity of the culture medium unlike other bacterial contamination. As a consequence, mycoplasmal infection of cell cultures could often persist for long periods of time without being noticed and with no apparent cell damage [9]. However, contaminating mycoplasmas affect virtually every parameter within the cell culture system. For instance, alterations in growth characteristics, enzyme patterns, cell membrane composition, chromosomal abnormalities, and induction of cytopathogenic changes have been described [10–12]. In what respects to stem cells, it was demonstrated that *Mycoplasma sp.* contamination of murine embryonic stem cells reduces growth rate and viability and affects their pluripotent capacity [13].

The effectiveness in mycoplasmas eradication of several antibiotics have been demonstrated *in vitro* [14–16]. Among these antibiotics, some of the more commonly used are Plasmocin^TM^ and Ciprofloxacin. Both are well-established anti-*Mycoplasma sp.* reagents that are used to cure contaminated cell lines in as little as a two-week treatment (25 µg/ml Plasmocin^TM^ and 10 µg/ml Ciprofloxacin) [16–21]. Moreover, Plasmocin^TM^ was also used prophylactically to prevent *Mycoplasma sp.* infection at a concentration of 5 µg/ml [22,23].

It is important to consider the possible toxic effects of antibiotic treatments on stem cell culture, especially with regards to cytotoxicity, loss of special cellular characteristics and clonal selection. In this sense, it has been reported the occurrence of cell culture loss as a result of cell death in up to 11% of the cell lines treated with anti-*Mycoplasma sp.* antibiotics [14]. The effectiveness of anti-*Mycoplasma sp.* antibiotics and their possible side effects have not been fully studied yet on human pluripotent stem cells. Therefore, the aim of this work is to study whether contaminated hESCs can be cured with an anti-*Mycoplasma sp.* antibiotic treatment and, even more important, if curative and also prophylactic treatments affect maintenance of undifferentiated state, self-renewal, pluripotency, viability and growth of hESCs.

In the present study we found that Plasmocin^TM^ curative treatment effectively eradicated *Mycoplasma sp.* contamination from the hESCs line HUES-5 884 (H5 884). Importantly, in cured H5 884 cells, stemness characteristics (cells morphology and expression of specific markers), karyotype and pluripotent capacity remained unaltered compared to HUES-5 (H5) parental cell line. Moreover, neither maintenance of the undifferentiated state nor pluripotent properties of hESCs lines WA09 (H9) and H5 were affected upon Plasmocin^TM^ and Ciprofloxacin prophylactic and curative treatments, respectively. However, even though anti-*Mycoplasma sp.* antibiotics did not affect hESCs viability or apoptosis rate, Plasmocin^TM^ and Ciprofloxacin slowed down hESCs growth rates when used at curative concentrations.

## Materials and Methods

### Reagents, cell lines and culture

Ciprofloxacin was purchased from Sigma (MO, USA) and Plasmocin^TM^ from Invivogen (CA, USA). The hESC line WA09 (H9) (46,XX karyotype) [1] was purchased from WiCell Research Institute (WI), and the hESC line HUES-5 (H5) [46,XX, inv(9) karyotype] [24] was acquired from Harvard University and the Howard Hughes Medical Institute (MA). Both cell lines are approved for US National Institute of Health (NIH) funding. hESCs were maintained on inactivated mouse embryonic fibroblast (iMEF) feeder layers in medium DMEM/F12 supplemented with 10% KSR, 2 mM non-essential amino acids, 2 mM L-glutamine, 100 U/ml penicillin, 50 µg/ml streptomycin, 0.1 mM β-mercaptoethanol and 4 ng/ml of bFGF. All these reagents were obtained from Invitrogen (CA, USA). hESCs were transferred with 1mg/ml collagenase IV (Invitrogen, CA, USA) into new iMEF feeder layers or into feeder-free diluted (1/40) Matrigel^TM^ (BD Bioscience, CA, USA) coated dishes containing iMEF conditioned medium. For the conditioning medium, 3x10^6^ iMEF cells were incubated for 24h with 25 ml of DMEM/F12 medium supplemented with 5% KSR and 2 ng/ml of bFGF (in addition to the other aforementioned supplements) and stored at -20°C. After thawing, KSR and bFGF were added to a final concentration of 20% and 8 ng/ml, respectively. For some experiments, hESCs grown on Matrigel^TM^ were dissociated into single cells using accutase 1x (Invitrogen, CA, USA) for 20 minutes, plated onto Matrigel^TM^ coated dishes (with addition of 10µM Y-27632 ROCK inhibitor, R&D Systems, MN, USA) and grown until confluence with conditioning medium.

Human foreskin fibroblasts were prepared as primary cultures from freshly obtained human foreskins as soon as possible after surgery. The isolated fibroblasts were then expanded, frozen and stored as described elsewhere. The study was given ethical approval by the local Ethics Committee (*Comité de ética* en investigaciones biomédicas del Instituto *FLENI*) and written informed consent was obtained from donor prior to fibroblast isolation.

### HUES-5 (H5)-Brachyury-GFP stable hESC cell line generation

The stable H5 cell line (H5 884) encoding GFP under the control of a fragment of the Brachyury promoter was generated as previously described [25].

### *Mycoplasma sp.* detection

Briefly, cells were washed with PBS and then scraped into 10 ml medium. 1 ml of cell suspension was transferred into a 1.5 ml eppendorf tube and centrifuged at 10,000 x g for 10 min at 4^°^C. Supernatant was discarded and pellet was suspended in 100 µl lysis buffer [10 mM Tris-HCl (pH 8.3), 50 mM KCl, 2 mM MgCl_2_, 0.001% gelatin, 0.5% NP-40, 0.5% Tween-20 and 1 µl of proteinase-K (10 mg/ml, Invitrogen, CA, USA)]. Cell suspension was lysed at 60^°^C for 1 hour. Nucleic acid was precipitated with 600 µl isopropanol, kept at -20^°^C for 30 min and centrifuged for 30 min at 4^°^C. Pellet was washed with 70% ethanol, air dried and re-suspended in 50 µl TE (10 mM Tris-HCl-1 mM EDTA pH 8.0). DNA content was determined using a NanoDrop 2000 Spectrophotometer (Thermo Scientific, MA, USA). 100 ng of sample DNA and 1 ng of positive control (genomic DNA from *Mycoplasma* *orale* ) were used for PCR amplification at an annealing temperature of 55^°^C. Primers used were: MYCPL; sense, 5´-ACACCATGGGAGYTGGTAAT-3´; antisense, 5´-CTTCWTCGACTTYCAGACCCAAGGCAT-3´.

### Embryoid bodies differentiation protocol

To induce differentiation, hESCs colonies were dispersed with 1 mg/ml collagenase IV (Invitrogen, CA, USA) for 1 hour. Cells were then transferred to non-adherent Petri dishes containing DMEM supplemented with 20% fetal bovine serum (Gibco, CA, USA), 2 mM L-glutamine, 100 U/ml penicillin and 50 µg/ml streptomycin used as differentiation medium. Cells incubated in suspension at 37^°^C and 5% CO_2_ for 7 days aggregated to form embryoid bodies (EBs), which were then plated onto 0.1% gelatin coated 24-well plates and cultured for additional 7 days. Normally, within 2-4 days after plating, tissue like-structures including contractile areas and neural rosettes were observed in the outgrowth of the EBs.

### RNA isolation and RT-qPCR

Total RNA was extracted from hESCs with Trizol (Invitrogen, CA, USA) and cDNA was synthesized from 500 ng of total RNA with 15 mM of random hexamers (Invitrogen, CA, USA) and MMLV reverse transcriptase (Promega, WI, USA), according to manufacturer’s instructions. For real-time PCR studies, cDNA samples were diluted 5-fold and PCR amplification and analysis were performed with StepOnePlus Real Time PCR System (PE Applied Biosystems, CA, USA). The SYBR^®^ GreenER^TM^ qPCR SuperMix UDG (Invitrogen, CA, USA) was used for all reactions, following manufacturer instructions. Primers used were the following: RPL7; sense, 5´-AATGGCGAGGATGGCAAG-3´; antisense, 5´-TGACGAAGGCGAAGAAGC-3´; OCT-4; sense, 5´-CTGGGTTGATCCTCGGACCT-3´; antisense, 5´-CACAGAACTCATACGGCGGG-3´; NANOG; sense, 5´-AAAGAATCTTCACCTATGCC-3´; antisense, 5´-GAAGGAAGAGGAGAGACAGT-3´; PAX-6; sense, 5´-CAGGTGTCCAACGGATGTG-3´; antisense, 5´-GTCGCTACTCTCGGTTTACTAC-3´; BRACHYURY; sense, 5´-TCCCAGGTGGCTTACAGATGA-3´; antisense, 5´-GGTGTGCCAAAGTTGCCAAT-3´; NKX2.5; sense, 5´-CCCACGCCCTTCTCAGTCAA-3´; antisense, 5´-GTAGGCCTCTGGCTTGAAGG-3´; α-FETOPROTEIN; sense, 5´-TGCTGGATTGTCTGCAGGATG-3´; antisense, 5´-ACGTTCCAGCGTGGTCAGTTT-3´.

### Immunofluorescence staining

hESCs were analyzed for *in situ* immunofluorescence. Briefly, cells were rinsed with PBS and fixed in PBSA (PBS with 0.1% bovine serum albumin) with 4% formaldehyde for 45 min. After two washes cells were permeabilized with 0.1% Triton X-100 in PBSA with 10% normal goat serum for 30 min, washed twice and stained with the corresponding primary antibodies: murine monoclonal antibodies anti-SSEA4 (813-70) (sc-21704), anti-Tra-1-60 (sc-21705), anti-Tra-1-81 (sc-21706), anti-Oct-3/4 (C-10) (sc-5279), anti-Troponin T-C (CT3) (sc-20025), anti-AFP (F8) (sc-166325) from Santa Cruz Biotechnology (CA, USA); rabbit monoclonal antibody anti-Nanog (D73G4) XP (R) from Cell Signaling Technology Inc. (MA, USA) and rabbit polyclonal antibody anti-Nestin (AB5922) from Millipore (MA, USA). Appropriate Alexa-conjugated secondary antibodies were purchased from Invitrogen (CA, USA) and used to localize the antigen/primary antibody complexes. Cells were counterstained with 4,6-diamidino-2-phenylindole (DAPI) (Invitrogen, CA, USA) and examined under a Nikon Eclipse TE2000-S inverted microscope. Images were acquired with a Nikon DXN1200F digital camera, which was controlled by the EclipseNet software (version 1.20.0 build 61). Cell number was estimated using the ITCN ImageJ-plugin software (Center for Bio-Image Informatics at University of California, USA) (Wayne Rasband, National Institutes of Health, USA. http://rsb.info.nih.gov/ij/).

### Cell viability assay

For cell viability assays, 1x10^4^ hESCs were plated onto Matrigel^TM^ coated 96-well plates and grown until confluence. 24 and 48h post antibiotics addition, 50 µg/well of activated 2,3-bis (2-methoxy-4-nitro-5-sulfophenyl)-5 [(phenylamino) carbonyl]-2-H-tetrazolium hydroxide (XTT) (Sigma, MO, USA) in PBS containing 0.3 µg/well of the intermediate electron carrier, N-methyl dibenzopyrazine methyl sulfate (PMS) (Sigma, MO, USA) were added (final volume 100 µl) and incubated for 2 h. Cellular metabolic activity was determined by measuring the absorbance of the samples with a spectrophotometer (Bio-Rad, CA, USA) at a wavelength of 450 nm and subtracting the background absorbance at 690 nm.

### TUNEL assay

An *in situ* cell death detection kit using TdT-mediated fluorescein-conjugated dUTP nick end labeling (TUNEL, In Situ Cell Death Detection Kit Fluorescein, AP; Roche Molecular Biochemicals, Mannheim, Germany) was used to detect apoptotic cells following manufacturer’s instructions. The procedure, using a photometric enzyme immunoassay, determines cytoplasmic histone-associated DNA fragments (mono- and oligonucleosomes) after cell death. Fluorescein labels were detected by fluorescence and examined under a Nikon Eclipse TE2000-S inverted microscope equipped with a 20X E-Plan objective and a super high-pressure mercury lamp. The images were acquired with a Nikon DXN1200F digital camera, which was controlled by the EclipseNet software (version 1.20.0 build 61). Cell number was estimated using the ITCN ImageJ-plugin software (Center for Bio-Image Informatics at University of California, USA) (Wayne Rasband, National Institutes of Health, USA. http://rsb.info.nih.gov/ij/).

### Alkaline Phosphatase assay

H9 hESCs were plated as small clumps onto iMEF feeder cells in 6-well plates. After 7 days antibiotics treatment, cells were washed with PBS and subjected to alkaline phosphatase staining following manufacturer’s instructions (Sigma, MO, USA).

### In-Cell Western assay

The assay was performed as per manufacturer instructions (Licor Bioscience, Lincoln, NE, USA). For cell growth experiments, small clumps of H9 were plated onto iMEF feeder layers 24-well plates and treated for 7 days. For cell growth recovery experiments, antibiotics were withdrawn after curative treatments, cells passaged twice and then same number of small clumps were plated onto iMEF feeder layers 24-well plates and cultured for 7 days. Cells were then rinsed with PBS and fixed in PBSA (PBS with 0.1% bovine serum albumin) with 4% formaldehyde for 45 min. After two washes cells were permeabilized with 0.1% Triton X-100 in PBSA with 10% normal goat serum for 30 min, washed twice and incubated overnight at 4^°^C with the corresponding primary antibodies: murine monoclonal antibody anti-Oct-3/4 (C-10) (sc-5279) 1:200 from Santa Cruz Biotechnology (CA, USA) and rabbit monoclonal antibody anti-Nanog (D73G4) XP (R) 1:400 from Cell Signaling Technology Inc. (MA, USA). After primary antibody incubations, plates were washed thrice with PBSA. Then, secondary antibodies from Licor Biosciences (NE, USA) conjugated to IRDye 800CW (goat-anti-mouse-IgG) 1:1,000 and to IRDye 680 (goat-anti-rabbit-IgG) 1:200 were used. Plates were incubated with secondary antibodies solutions for 60 minutes at room temperature in the dark. Background control wells were prepared by omitting primary antibodies (i.e., secondary only). After secondary antibody incubations, plates were washed thrice with PBSA the dark, and then plates were air-dried before scanning. Plates were scanned and analyzed using an Odyssey Infrared Imaging Scanner and Odyssey imaging software 3.0. Scan settings used were medium image quality, 169 µm resolution, intensity 5.0 for the both the 700-channel and 800-channel with an offset of 3.0 mm. For signal quantification, antibody signals were analyzed as the average 700 and 800-channel integrated intensities from triplicate wells. Background signal was subtracted.

### Statistical analysis

Results were expressed as Means ± S.E. One-way ANOVAs followed by Turkey’s multiple comparisons test were used to detect significant differences among treatments. Statistical analyses were performed with STATISTICA 6.0 (StatSoft, Inc.). Differences were regarded as significant at *p*<0.05.

## Results

### Analysis of cell morphology, stem cell markers expression and karyotype in *Mycoplasma sp.* infected human embryonic stem cells cured with Plasmocin^TM^ antibiotic

**Figure 1:**
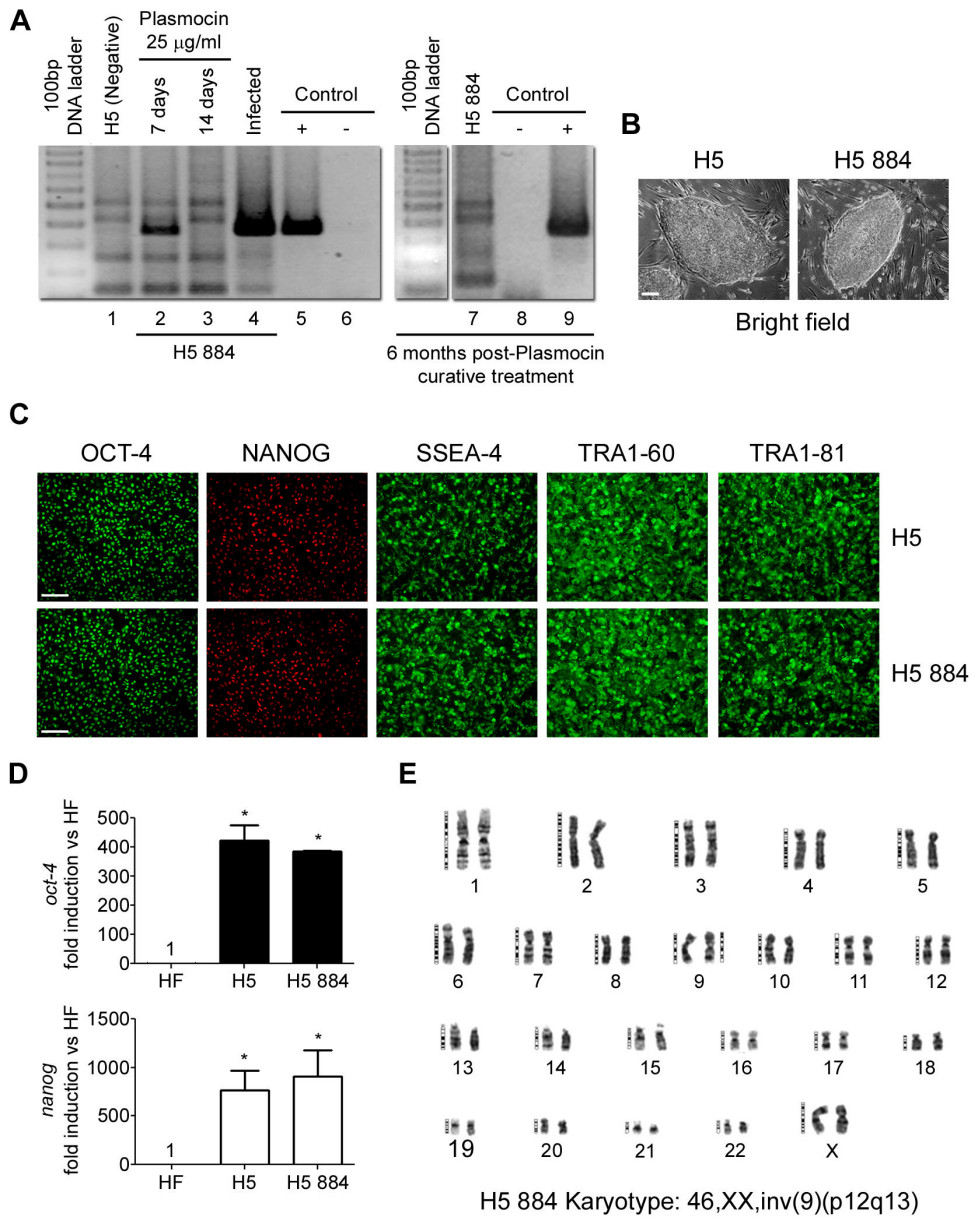
Infected hESCs cured with Plasmocin^TM^ maintain their morphology, stem cell markers expression and karyotype. *Mycoplasma sp.* infected HUES-5 884 (H5 884) cells were treated with Plasmocin 25 µg/ml during 14 days (Curative treatment) and: (A) *Mycoplasma sp.* infection was followed by genomic DNA PCR analysis at day 0, 7 and 14 after antibiotic addition (lanes 4, 2 and 3), in non infected parental H5 line (lane 1) and in cured cells after 6 month post-treatment (lane 7) Control +: *Mycoplasma* *orale* genomic DNA (lanes 5 and 9). Control -: H_2_O (lanes 6 and 8); (B) Cured H5 884 cells were photographed using an inverted microscope in order to compare to HUES-5 (H5) colony morphology. Figure shows representative bright field photomicrographs. Scale bars = 100 µm; (C) H5 and cured H5 884 cells were grown on Matrigel^TM^ coated plates until confluence and stained with primary antibodies that recognize stem cell markers. Figure shows representative fluorescent photomicrographs of hESCs immunostained with SSEA-4, TRA-1-60, TRA-1-81, Nanog and Oct-4. Scale bars = 100 µm; (D) H5 884 mRNA levels of *oct-4* and *nanog* were analyzed by RT-Real Time PCR after 14 days of curative treatment and compared to H5 levels. *Rpl7* expression was used as normalizer. Graph shows mRNA fold induction relative to human fibroblasts (HF). The mean ± S.E. from three independent experiments are shown. * = *p*<0.05; (E) Karyotype analysis of cured H5 884 cells. A representative GTG-banded metaphase spread is shown. Abbreviations: SSEA-4, Stage-Specific Embryonic Antigen; TRA-1-60, Tumor Rejection Antigen 1-60; TRA-1-81, Tumor Rejection Antigen 1-81; Oct-4, Octamer 4.

A stable HUES-5 (H5) hESC line encoding GFP gene under the control of a fragment of the Brachyury promoter (H5 884) was developed for mesoderm-based studies. Unfortunately, after months of work and before being cryopreserved (initial stock), *Mycoplasma sp.* contamination was detected in this cell line by PCR analysis (Figure 1A, lane 4). As this culture was considered an extremely valuable tool, we decided to try to eradicate *Mycoplasma sp.* contamination. For this purpose, H5 884 cells were treated with Plasmocin 25 µg/ml during 14 consecutive days (curative treatment). As shown in Figure 1A, after 7 days antibiotic treatment (lane 2), positive PCR band decreased. Moreover, after 14 days treatment (lane 3), *Mycoplasma sp.* genomic DNA was not amplified. Importantly, 6 months post-Plasmocin^TM^ curative treatment *Mycoplasma sp.* continued undetected on H5 884 cells, which means that cells remained free of mycoplasmas over long-term culturing (Figure 1A, lane 7). These results indicate that we were able to permanently eradicate *Mycoplasma sp.* infection from H5 884 cells.

We next tested if Plasmocin^TM^ curative treatment affected undifferentiated status, self-renewal and karyotype of H5 884 hESCs. After antibiotic curative treatment, H5 884 cells morphology was compared to H5 cells. As it can be seen in Figure 1B, H5 884 cells retained hESCs morphological characteristics, like formation of compact multicellular colonies with a high nucleus/cytoplasm ratio, prominent nucleoli and distinct colony borders.

Furthermore, H5 and cured H5 884 cells were grown until confluence on Matrigel^TM^ coated culture dishes and then stem cells associated markers expression was analyzed by immunofluorescence microscopy. Both H5 and H5 884 cells exhibited robust expression of different stemness markers, such as the nuclear located transcription factors Oct-4 and Nanog and the surface markers SSEA-4, TRA1-60 and TRA1-81. No areas of lack of expression of stemness markers were detected (Figure 1C). Moreover, tested cell lines also expressed significant high levels of stemness associated transcripts, quantified by RT-Real time PCR, such as *oct-4* and *nanog*, compared to human fibroblasts (Figure 1D). Besides, no significant difference was observed between H5 884 and H5 cells.

As previously mentioned, both *Mycoplasma sp.* contamination and antibiotic treatment may cause chromosomal abnormalities. Interestingly, after mycoplasmas eradication treatment, H5 884 cells exhibited the same karyotype [46,XX, inv(9) (p12q13)] (Figure 1E) as the parental H5 line [24].

All together, the above results show that Plasmocin^TM^ curative anti-*Mycoplasma sp.* treatment did not affect hESCs undifferentiated state nor karyotype.

### Effect of Plasmocin^TM^ anti-*Mycoplasma sp.* curative treatment on human embryonic stem cells pluripotency

**Figure 2:**
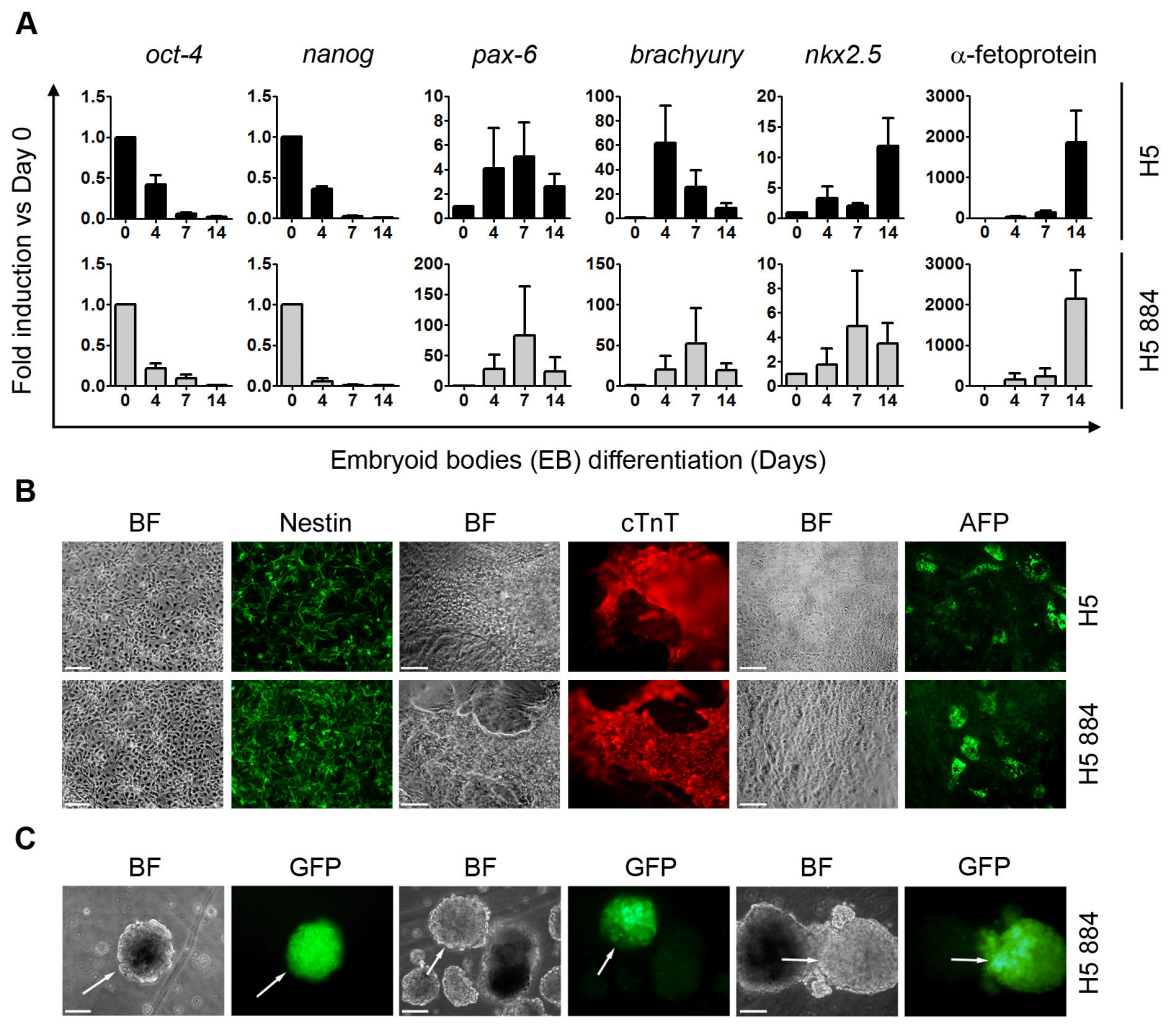
Differentiation of hESCs cured with Plasmocin^TM^. H5 884 cells treated with Plasmocin^TM^ 25 µg/ml during 14 days (Curative treatment) and H5 cells were differentiated using the EBs protocol and then: (A) mRNA levels of *oct-4* and *nanog*, ectoderm (*pax-6*), mesoderm (*brachyury* and *nkx2.5*) and endoderm (α-fetoprotein) markers were analyzed by RT-Real Time PCR at days 0, 4, 7 and 14 of the differentiation protocol. *Rpl7* expression was used as normalizer. Graph shows mRNA fold induction relative to day 0. The mean ± S.E. from three independent experiments are shown. (B) Cells were grown on gelatin coated plates from day 7 to 14 of differentiation and then ectoderm (neural rosettes), cardiac mesoderm (contractile EBs) and endoderm structures were stained with primary antibodies that recognize Nestin (ectoderm), cTnT (cardiac mesoderm) and AFP (endoderm) markers. Figure shows representative images. Scale bars = 100 µm. (C) Cured H5 884 cells, which express GFP gene under the control of the Brachyury promoter, were differentiated using the EBs protocol. At day 4 of the onset of differentiation GFP green fluorescence was photographed using an inverted fluorescence microscope. Figure shows representative images. Arrows indicate GFP positive regions or EBs. Scale bars = 100 µm. Abbreviations: BF, Bright field; Pax-6, Pair box protein 6; Nkx2.5, NK2 homeobox 5; cTnT, Cardiac troponin T; AFP, Alpha-fetoprotein; GFP, Green fluorescent protein.

Based on our observation that *Mycoplasma sp.* infected hESCs cured with Plasmocin^TM^ antibiotic retained their stemness, we asked if this curative treatment affected hESCs pluripotent potential. *In vitro* differentiation of hESCs can be achieved by removing cells from feeders layers and growing them in suspension (with the addition of serum which contains all necessary differentiation signals) on a non-adherent surface [26]. Under these conditions, pluripotent hESCs form three-dimensional multicellular aggregates called embryoid bodies (EBs) and differentiate into any cell lineage of the three germ layers. Importantly, cured H5 884 cells responded to the differentiation protocol in a similar way to H5 cells. In this sense, mRNA levels of *oct-4* and *nanog*, diminished at days 4, 7 and 14 of the onset of differentiation (Figure 2A). At the same time points, mRNA levels of differentiation markers, such as *pax-6* (ectoderm), *brachyury* (early mesoderm), *nkx2.5* (cardiac mesoderm) and α-fetoprotein (endoderm), were up-regulated (Figure 2A). Moreover, on day 14 of the differentiation protocol (after 7 days in suspension followed by 7 days of outgrowth in gelatin coated-dishes) we observed formation of neuro-ectoderm, endoderm and cardiac contractile regions, positively stained for Nestin, α-Fetoprotein (AFP) and cardiac Troponin T (cTnT), respectively (Figure 2B).

As previously mentioned, stable H5 884 cell line encodes GFP gene under the control of a fragment of the Brachyury promoter. Brachyury is an early mesoderm marker whose expression peaks at day 4 of the EBs-based differentiation protocol. We were able to detect GFP green fluorescence (indicative of Brachyury promoter up-regulation) in some H5 884 EBs at day 4 of the onset of differentiation (Figure 2C). These observations confirmed that Plasmocin^TM^ curative treatment did not impair hESCs pluripotent potential.

#### Impact of Ciprofloxacin anti-*Mycoplasma sp.* "curative" treatment on human embryonic stem cells cell morphology, stem cells markers expression and pluripotency

**Figure 3:**
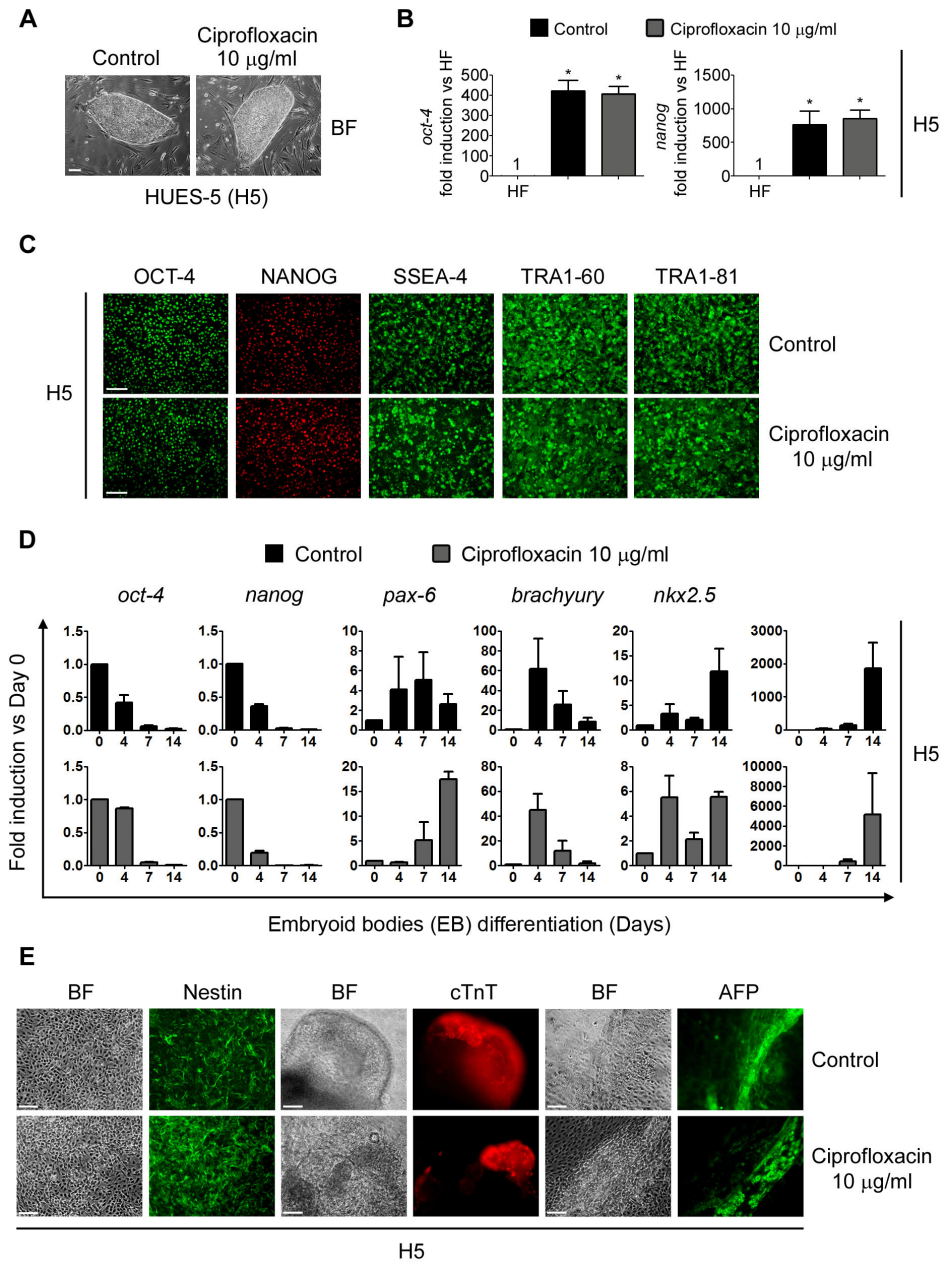
hESCs treated with Ciprofloxacin maintain their morphology, stem cell markers expression and pluripotency. H5 cells were treated with Ciprofloxacin 10 µg/ml during 14 days (Curative treatment mimic) and then: (A) photographed using an inverted microscope in order to compare colony morphology. Control: untreated cells. Figure shows representative bright field photomicrographs. Scale bars = 100 µm; (B) mRNA levels of the stemness markers *oct-4* and *nanog* were analyzed by RT-Real Time PCR after 14 days of "curative" treatment. *Rpl7* expression was used as normalizer. Control: untreated cells. Graph shows mRNA fold induction relative to human fibroblasts (HF). The mean ± S.E. from three independent experiments are shown. * = *p*<0.05; (C) cells were grown on Matrigel^TM^ coated plates until confluence and stained with primary antibodies that recognize stem cell markers. Control: untreated cells. Figure shows representative fluorescent photomicrographs of hESCs immunostained with SSEA-4, TRA-1-60, TRA-1-81, Nanog and Oct-4. Scale bars = 100 µm; (D) mRNA levels of stemness (*oct-4* and *nanog*), ectoderm (*pax-6*), mesoderm (*brachyury* and *nkx2.5*) and endoderm (α-fetoprotein) markers were analyzed by RT-Real Time PCR at days 0, 4, 7 and 14 of the EBs differentiation protocol. *Rpl7* expression was used as normalizer. Control: untreated cells. Graph shows mRNA fold induction relative to day 0. The mean ± S.E. from three independent experiments are shown. (E) Cells were grown on gelatin coated plates from day 7 to 14 of differentiation and then ectoderm (neural rosettes), cardiac mesoderm (contractile EBs) and endoderm structures were stained with primary antibodies that recognize Nestin (ectoderm), cTnT (cardiac mesoderm) and AFP (endoderm) markers. Figure shows representative images. Scale bars = 100 µm.

H5 cells were treated with Ciprofloxacin 10 µg/ml during 14 days in order to mimic a curative treatment. After antibiotic treatment, cells retained hESCs morphological characteristics compared to control untreated cells (Figure 3A).

After Ciprofloxacin "curative" treatment, H5 stem cells associated markers expression was analyzed by RT-Real Time PCR and immunofluorescence microscopy. As occurred with Plasmocin^TM^ curative treatment, Ciprofloxacin-treated and untreated cells robustly express stemness markers at *both mRNA and protein levels* (Figure 3C).

Maintenance of H5 pluripotent potential after Ciprofloxacin "curative" treatment was then tested. Importantly, treated H5 hESCs responded to the EBs differentiation protocol in a similar way to untreated cells. Again, mRNA levels of *oct-4* and *nanog*, quantified by RT-Real time PCR, diminished at days 4, 7 and 14 of the onset of differentiation (Figure 3D). At the same time points, mRNA levels of *pax-6*, *brachyury*, *nkx2.5* and α-fetoprotein were up-regulated (Figure 3D). Moreover, on day 14 of the differentiation regions of Nestin, AFP or cTnT-positive cells were identified by immunocytochemistry (Figure 3E). All together, the above results show that Ciprofloxacin anti-*Mycoplasma sp.* "curative" treatment did not affect hESCs undifferentiated state nor pluripotency.

### Plasmocin^TM^ prophylactic anti-*Mycoplasma sp.* treatment: implications on human embryonic stem cells special characteristics

**Figure 4:**
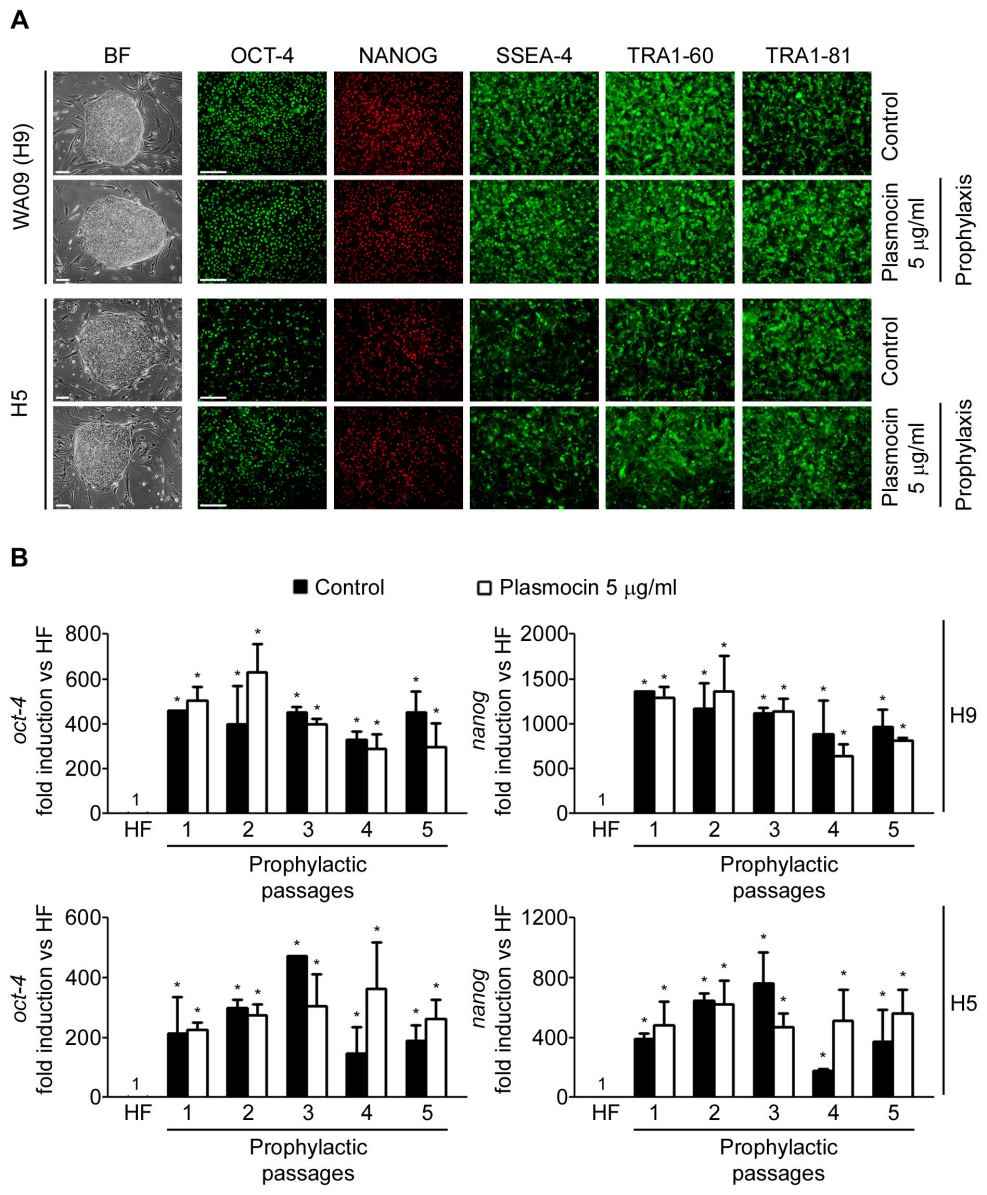
hESCs maintain their morphology and stem cell markers expression upon Plasmocin^TM^ prophylactic treatment. WA09 (H9) and H5 cells were treated with Plasmocin^TM^ 5 µg/ml during 5 consecutive passages (one passage per week) (Prophylactic treatment) and then: (A) photographed using an inverted microscope in order to compare colony morphology; and grown on Matrigel^TM^ coated plates until confluence and stained with primary antibodies that recognize stem cell markers. Control: untreated cells. Figure shows representative bright field and fluorescent images of hESCs immunostained or not with antibodies against SSEA-4, TRA-1-60, TRA-1-81, Nanog and Oct-4. Scale bars = 100 µm; (B) mRNA levels of *oct-4* and *nanog* were analyzed by RT-Real Time PCR on each passage of the 5 consecutive passages of the prophylactic treatment. Control: untreated cells. *Rpl7* expression was used as normalizer. Graph shows mRNA fold induction relative to human fibroblasts (HF). The mean ± S.E. from three independent experiments are shown. *=p<0.05.

hESC lines H9 and H5 were treated with Plasmocin^TM^ 5 µg/ml during 5 consecutive passages (prophylactic treatment). Importantly, post-prophylactic treatment cells retained hESCs morphological characteristics and stem cells associated markers expression (analyzed by RT-Real Time PCR and immunofluorescence) compared to control untreated cells (Figure 4A and B). Moreover, H9 and H5 treated and control untreated cells exhibited robust expression of Oct-4, Nanog, SSEA-4, TRA1-60 and TRA1-81 (Figure 4A). Besides, *oct-4* and *nanog* transcripts were significantly highly expressed in H9 and H5 treated cells compared to human fibroblasts (Figure 4B). Despite the expected variability observed between different passages, no significant differences in either *oct-4* or *nanog* expression was observed between treated (prophylactic) and control cells (Figure 4B).

**Figure 5:**
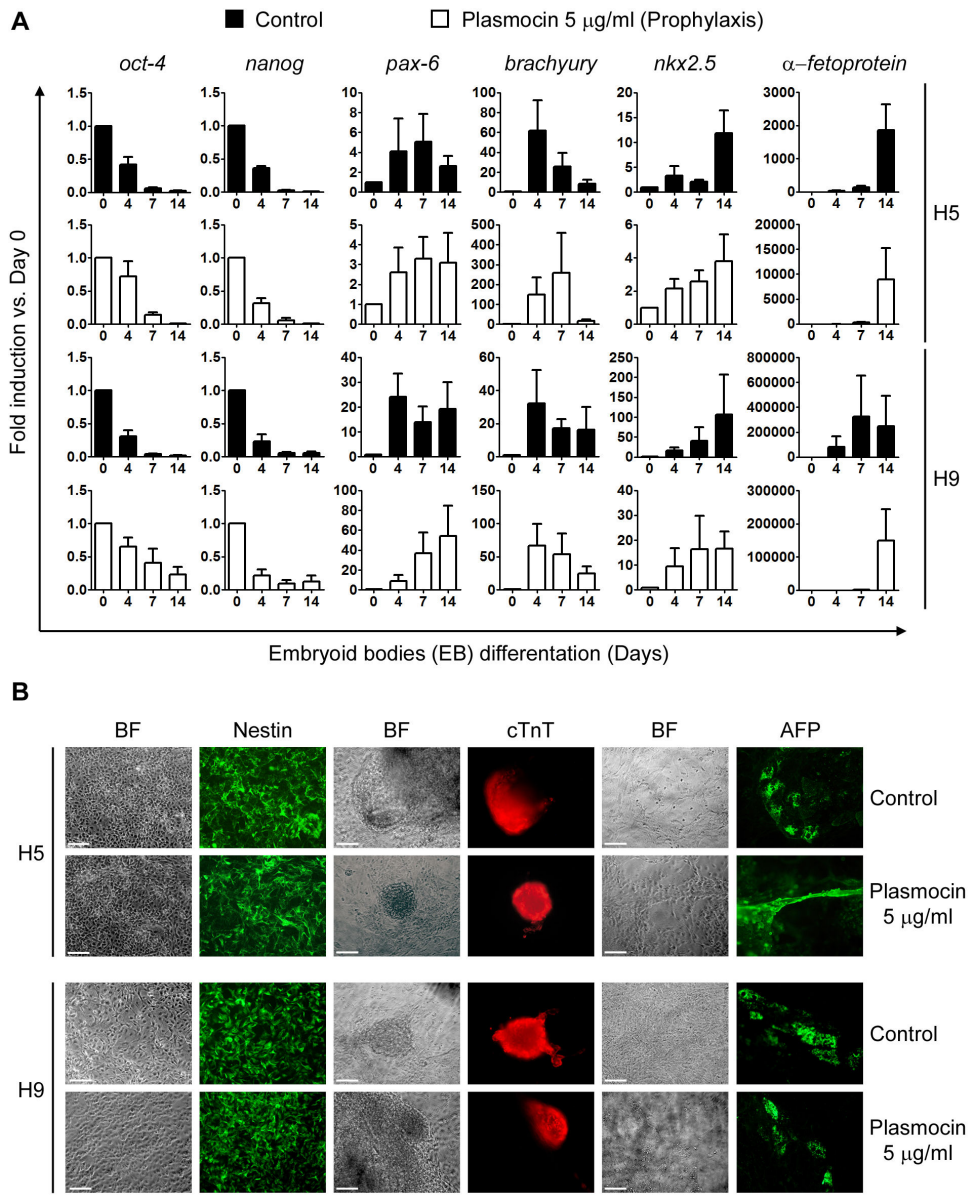
Differentiation of hESCs upon Plasmocin^TM^ prophylactic treatment. H5 and H9 cells treated with Plasmocin^TM^ 5 µg/ml during 5 consecutive passages (Prophylactic treatment) were differentiated using the EBs protocol and then: (A) mRNA levels of stemness (*oct-4* and *nanog*), ectoderm (*pax-6*), mesoderm (*brachyury* and *nkx2.5*) and endoderm (α-fetoprotein) markers were analyzed by RT-Real Time PCR at days 0, 4, 7 and 14 of the differentiation protocol. *Rpl7* expression was used as normalizer. Control: untreated cells. Graph shows mRNA fold induction relative to day 0. The mean ± S.E. from three independent experiments are shown. (B) Cells were grown on gelatin coated plates from day 7 to 14 of differentiation and then ectoderm (neural rosettes), cardiac mesoderm (contractile EBs) and endoderm structures were stained with primary antibodies that recognize Nestin (ectoderm), cTnT (cardiac mesoderm) and AFP (endoderm) markers. Control: untreated cells. Figure shows representative images. Scale bars = 100 µm.

We next wondered if Plasmocin^TM^ prophylactic treatment affects H9 and H5 hESCs pluripotent potential. Treated and control hESCs H5 and H9 were differentiated using the EBs differentiation protocol. Once again, H5 and H9 hESCs responded equally to the EBs differentiation protocol as judged by RT-Real time PCR analysis of stemness and differentiation markers mRNAs levels and immunofluorescence microscopy with antibodies against Nestin, AFP and cTnT (Figure 5A and B). We can conclude from these experiments that Plasmocin^TM^ prophylactic treatment against *Mycoplasma sp.* infection did not altered hESCs special characteristics, like stemness properties and pluripotency.

### Cytotoxicity of Plasmocin^TM^ and Ciprofloxacin antibiotics on hESCs viability, apoptosis rate and growth

**Figure 6:**
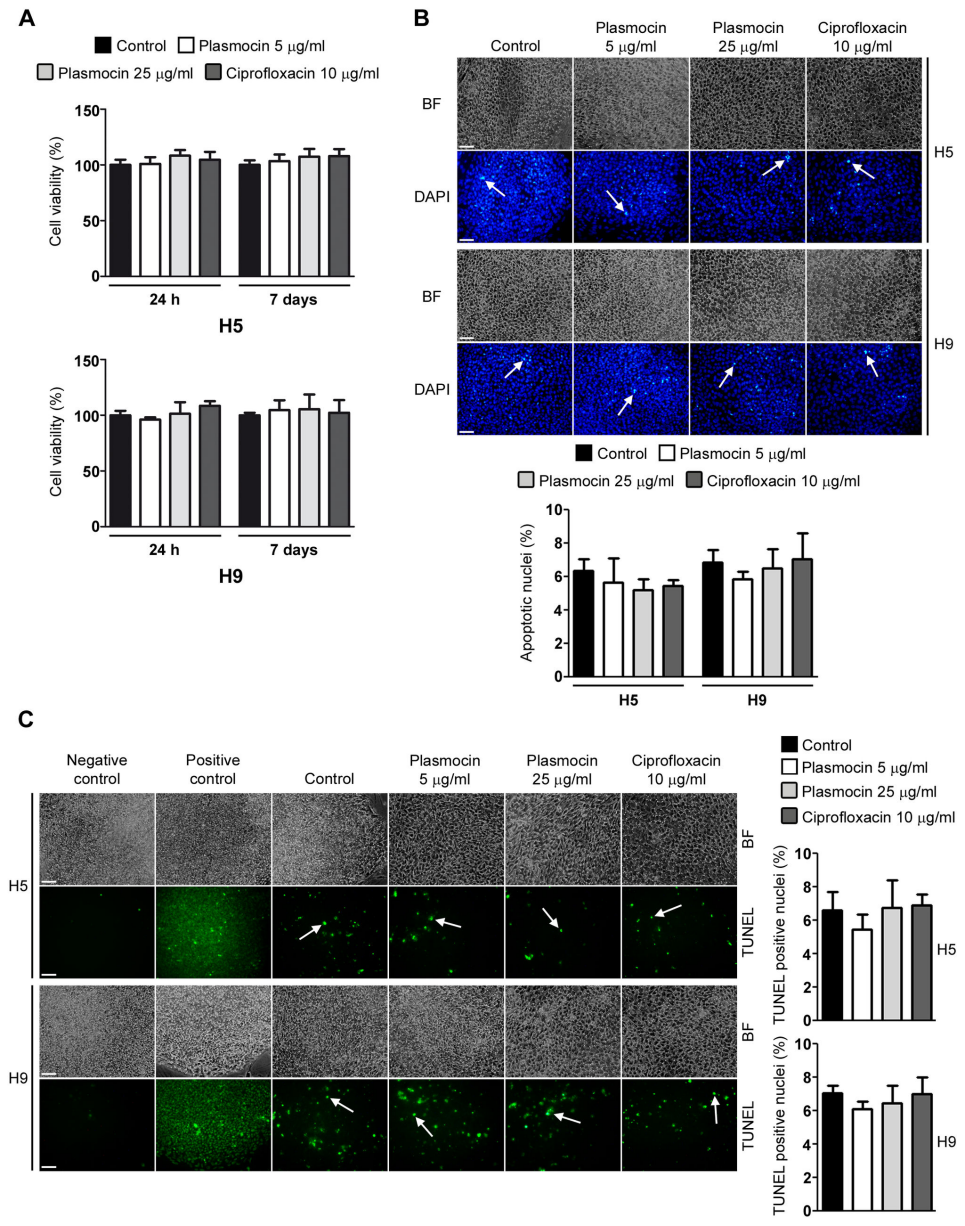
Cell viability and apoptosis rate of hESCs treated with Plasmocin^TM^ and Ciprofloxacin. (A) Cell viability was assessed by XTT colorimetric assay performed at 24 h and 7 days post Plasmocin^TM^ (5 or 25 µg/ml) or Ciprofloxacin (10 µg/ml) treatments on feeder-free confluent H5 and H9 cells. The results are presented as percentage of control untreated cells. Bars indicate mean ± S.E. of four replicates from three independent experiments (n=12). * = *p*< 0.05. (B) Chromatin condensation was analyzed by DAPI staining of H5 and H9 cells grown on iMEF feeder layer and treated with Plasmocin^TM^ (5 or 25 µg/ml) or Ciprofloxacin (10 µg/ml) for 7 days. Figure shows representative images and arrows indicate apoptotic nuclei. Mean ± S.E. from three independent experiments are graphed for % of apoptotic nuclei. Scale bars = 100 µm. * = *p*<0.05. (C) DNA fragmentation was assessed by TUNEL assay performed at 7 days post Plasmocin^TM^ (5 or 25 µg/ml) or Ciprofloxacin (10 µg/ml) treatments on H5 and H9 cells grown on iMEF feeder layer and is expressed as percentage of TUNEL positive nuclei. Control: untreated cells. Positive control: control cells treated with DNAse I. Negative control: control cells in label solution only (without terminal transferase). Representative images are shown and arrows indicate TUNEL positive nuclei. Mean ± S.E. from three independent experiments are graphed. Scale bars = 100 µm. *=p<0.05.

As previously mentioned, anti-*Mycoplasma sp.* antibiotics could have cytotoxic effects on cell viability. We next evaluated if Plasmocin^TM^ at 5 and 25 µg/ml and Ciprofloxacin at 10 µg/ml could affect cell viability of confluent H5 and H9 hESCs grown in feeder-free conditions. As shown in Figure 6A, after 24 and 7 days treatments, the percentage of viable cells did not significantly change. Importantly, neither Plasmocin^TM^ nor Ciprofloxacin at the concentrations tested decreased hESCs viability.

Chromatin condensation paralleled by DNA fragmentation are two of the most important criteria which are used to identify apoptotic cells. Therefore we next measured these processes after 7 days of Plasmocin^TM^ (5 or 25 µg/ml) or Ciprofloxacin (10 µg/ml) treatments on H5 and H9 cells by DAPI staining of nuclear DNA and TUNEL technique, respectively. We found that antibiotics treatment did not significantly increased the percentage of hESCs DAPI positive apoptotic nuclei (Figure 6B). Moreover, in what it concerns to DNA fragmentation, no significant increase in TUNEL-positive cells was found in H5 and H9 hESCs treated cells (Figure 6C). In conclusion, Plasmocin^TM^ and Ciprofloxacin treatments did not affect hESCs apoptosis rate.

**Figure 7:**
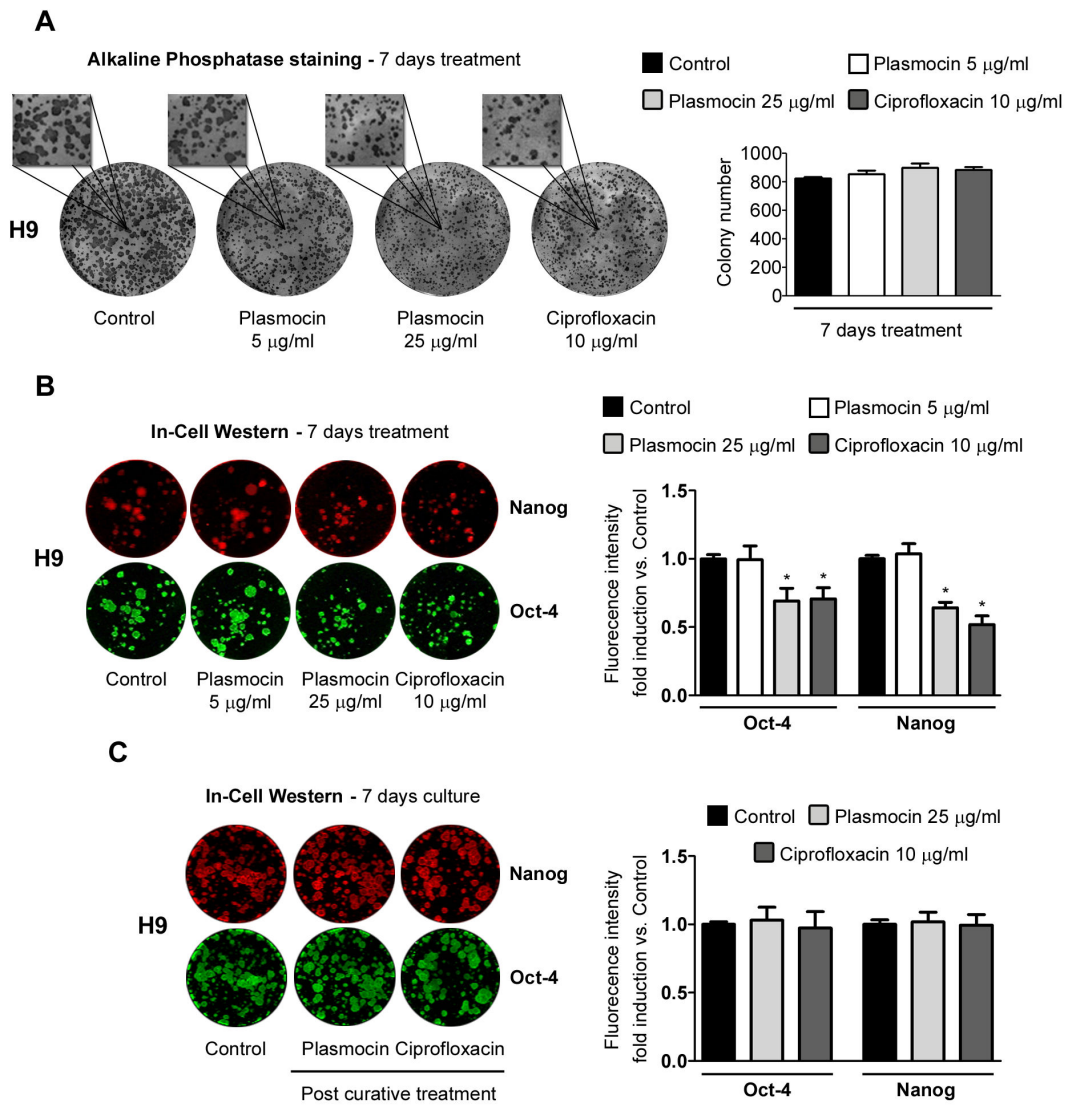
Cell growth of hESCs treated with Plasmocin^TM^ and Ciprofloxacin. (A) Alkaline phosphatase staining of H9 cells grown on iMEF feeder layer after 7 days of treatment with Plasmocin^TM^ (5 or 25 µg/ml) or Ciprofloxacin (10 µg/ml). Control: untreated cells. Representative images and 250% magnifications are shown. Colony number is graphed. Bars indicate mean ± S.E. of three independent experiments. * = *p*<0.05. (B) In-Cell Western analysis of Nanog and Oct-4 stemness markers on H9 cells grown on iMEF feeder layer after 7 days of treatment with Plasmocin^TM^ (5 or 25 µg/ml) or Ciprofloxacin (10 µg/ml). Control: untreated cells. Representative images are shown. Fluorescence intensity fold induction vs. Control is shown. Bars indicate mean ± S.E. of three independent experiments. * = *p*<0.05. (C) After 14 days Plasmocin^TM^ (25 µg/ml) or Ciprofloxacin (10 µg/ml) "curative" treatments, antibiotics were removed and H9 cells were passaged twice and then grown on iMEF feeder layer for 7 days under regular culture media conditions in order to study Nanog and Oct-4 stemness markers expression by In-Cell Western. Control: untreated cells. Representative images are shown. Fluorescence intensity fold induction vs. Control is shown. Bars indicate mean ± S.E. of three independent experiments. *=p<0.05.

Finally, we studied if antibiotic treatments affect hESCs growth. In order to answer this question, we plated small clusters of H9 cells on iMEF feeder layers and, after 7 days of Plasmocin^TM^ (5 and 25 µg/ml) and Ciprofloxacin (10 µg/ml) treatments, hESCs colonies size and number were analyzed. Qualitative observation of alkaline phosphatase staining (phenotypic marker of pluripotent stem cells) of H9 cells showed smaller colony size for antibiotic treated cells, with Plasmocin^TM^ 25 µg/ml and Ciprofloxacin 10 µg/ml, compared to control cells. However, total colony number remained unaltered (Figure 7A). Moreover, In-Cell Western analysis of H9 cells revealed significant lower fluorescent signals for Nanog and Oct-4 on Plasmocin^TM^ 25 µg/ml and Ciprofloxacin 10 µg/ml 7 days-treated cells (Oct-4: 0.69±0.09, 0.70±0.08; Nanog: 0.64±0.09, 0.51±0.15 fluorescence intensity fold change vs. control untreated cells, for Plasmocin^TM^ 25 µg/ml and Ciprofloxacin 10 µg/ml, respectively) (Figure 7B). This quantification perfectly correlates with the smaller colony size observed (Figure 7B). Importantly, after antibiotics withdrawal cell growth rate was recovered (Figure 7C).

Taken together, these results indicate that the anti-*Mycoplasma sp.* antibiotics tested herein did not affect cell viability or apoptosis rate. Nevertheless, Ciprofloxacin and Plasmocin^TM^ at curative concentrations (10 and 25 µg/ml, respectively) diminished cell growth.

## Discussion

hESCs cell therapies have been proposed for regenerative medicine and tissue replacement after injury or disease [1]. Besides these promising uses, hESCs are currently useful tools for early human development basic research, genetic disease modeling and *in vitro* systems for toxicology testing [27].

However, hESCs may harbor infectious agents, particularly *Mycoplasma sp.*, acquired during *in vitro* culture. The use of contaminated differentiated cells for cell replacement therapies increases the risk of transmitting infectious agents to human patients. In addition, alteration of host cellular characteristics caused by contaminating *Mycoplasma sp.*, can greatly influence the experimental results obtained with hESCs on research and *in vitro* toxicity test models and, therefore lead to misleading conclusions. Moreover, contamination can also result in economic setbacks because of the loss of time and, since *mycoplasmas* are hard to eradicate, the loss of precious cell lines. Taken these drawbacks into account, it is essential to extreme aseptic conditions and to emphasize the need to regularly screen cell cultures for mycoplasmal contamination. There are many different methods for the detection of *Mycoplasma sp.* contamination in cell cultures, as for example microbial culture expansion in broth culture and detection by colony formation on nutrient agar plates, DNA fluorochrome staining and biochemical assays [28]. However, infections can remain undetected unless more sensitive methods such as polymerase chain reaction (PCR) are employed. MEFs and hESCs lines cultured in this work were routinely tested for *Mycoplasma sp.* contamination by PCR, using *Mycoplasma sp*.-specific primer sequences [29]. Except for the infected H5 884 cells, in all cases cultures resulted free of contaminants.

Laboratory personnel, contaminated serum or reagents and other contaminated cell cultures are the major sources of *Mycoplasma sp.* infections [12,14,30]. Thus, positive cultures should be discarded and replaced in order to prevent the spreading of the contaminant. If the culture is considered irreplaceable, like recently generated hESC stable lines (our H5 884 hESC line for example) or induced pluripotent stem cell lines, it is possible to effectively eliminate the *Mycoplasma sp.* contamination. Obviously, methods of *mycoplasmas* eradication should be simple, have minimal effect on cell growth and not lead to loss of special cellular hESCs characteristics. The most simple and effective technique is antibiotic treatment and many different anti-*Mycoplasma sp.* reagents, including Plasmocin^TM^ and Ciprofloxacin, have been already used successfully in several different eukaryotic cell lines [12,14–16,21]. Nevertheless, changes in culture conditions of hESCs could lead to the loss of the ability of self-renewal, pluripotency and induction of differentiation. In this sense, understanding if anti-*Mycoplasma sp.* antibiotics could cure *Mycoplasma* sp.-infected hESCs, how they could affect these special properties and also if they exert cytotoxic effects over hESCs viability and growth is of growing interest in the search of better ways of controlling *Mycoplasma sp.* infections in hESCs culture facilities.

In the present study, we assessed for the first time the effect of the anti-*Mycoplasma sp.* antibiotics Plasmocin^TM^ and Ciprofloxacin on hESCs stemness, pluripotency, cell viability and growth. We used three different approaches: first, a 14-days curative treatment course with high concentrations of Plasmocin^TM^ (25 µg/ml) in order to cure hESCs (H5 884) infected with mycoplasmas; second, a 14-days treatment course with high concentrations of Ciprofloxacin (10 µg/ml) that mimics a treatment that could be used to eradicate *Mycoplasma sp.* infections ("curative" treatment); third, treatment with Plasmocin^TM^ at a concentration of 5 µg/ml during 5 consecutive passages in order to test if this antibiotic could be used to prevent from *Mycoplasma sp.* contamination (prophylactic treatment). After treatments, antibiotics were withdrawn and we tested if cells continued satisfying the criteria used to define a pluripotent state. We found that the expression profile of stemness markers and morphological characteristics in antibiotic prophylactic and curative-treated cells did not differ from control untreated or parental cells. Besides, karyotype of Plasmocin^TM^-cured H5 884 was still the same of H5 parental cells. Moreover, we were able to identify cell derivatives of the three embryonic germ layers in EBs derived from hESCs treated with Plasmocin^TM^ or Ciprofloxacin under both curative and prophylactic conditions.

The above results indicate that these cells are still pluripotent and though are capable to differentiate *in vitro*. Thus, Plasmocin^TM^ and Ciprofloxacin curative and prophylactic treatments did not affect hESCs differentiation characteristics and could be used to eradicate and prevent *Mycoplasma sp.* contamination on these cells. However, application of antibiotics in cell culture should be limited, as indiscriminate use could lead, among others, to the emergence of resistant *mycoplasmas* strains. To lessen this effect Plasmocin^TM^ contains two bactericidal components strongly active against *mycoplasmas*. The first component acts on the protein synthesis machinery while the second acts on the DNA replication. The combination of these two not related bactericidal components makes highly unlikely the appearance of resistant *Mycoplasma sp*. In contrast, Ciprofloxacin is a quinolone that only inhibits topoisomerase enzymes (relaxation of supercoiled DNA is affected promoting breakage of double stranded DNA), so prolonged use of this antibiotic on cell culture may lead to the selection of resistant *mycoplasmas*.

Anti-Mycoplasma *sp.* antibiotics could have cytotoxic effects over hESCs viability and growth. We found that neither Plasmocin^TM^ nor Ciprofloxacin at the concentrations studied affected H9 and H5 hESCs viability or apoptosis rate. In this sense, previous studies on *Mycoplasma sp.* free eukaryotic cell lines do not provide any evidence of antibiotic effect on host cell viability toxicity [31]. However, we observed that Plasmocin^TM^ and Ciprofloxacin at curative concentrations (25 and 10 µg/ml, respectively) significantly reduced H9 hESCs growth rate. Importantly, this effect was reversible, as growth rate was recovered after antibiotic withdrawal. On the other hand, the effect of Plasmocin^TM^ on cell growth rate at prophylactic concentrations (5 µg/ml) was minimum. Previous reports described inhibitory effects of Ciprofloxacin on hematopoietic progenitors cell growth [32,33]. Moreover, at high concentrations of Plasmocin^TM^ (like 25 µg/ml), slowdown of cell growth had been already observed. This slowing down was mainly due to the inhibition of mitochondrial respiration by Plasmocin^TM^ (manufacturer information).

In conclusion, Plasmocin^TM^ and Ciprofloxacin anti-*Mycoplasma sp.* antibiotics, at both curative and prophylactic treatment conditions, did not affect cell viability, maintenance of the undifferentiated state and pluripotency of hESCs. Proper cell culture aseptic practices along with specific, sensitive and reliable detection methods can provide an appropriate situation to prevent *Mycoplasma sp.* contamination in hESCs culture facilities. However, according to our findings, if an irreplaceable hESCs culture is contaminated, either of these antibiotics could be used to eradicate *mycoplasmas*. Moreover, Plasmocin^TM^ at prophylactic concentrations could be used, preferably during short periods of time, to prevent from *Mycoplasma sp.* contaminations in high-risk environments. Nevertheless, further experiments using define differentiation protocols should be perform to discard the possibility that lineage-restricted progenitors or terminally differentiated cell populations could be affected.
